# Patient outcome quality indicators for older persons in acute care: original development data using interRAI AC-CGA

**DOI:** 10.1186/s12877-024-04980-9

**Published:** 2024-06-17

**Authors:** Melinda G. Martin-Khan, Leonard C. Gray, Caroline Brand, Olivia Wright, Nancy A. Pachana, Gerard J. Byrne, Mark D. Chatfield, Richard Jones, John Morris, Catherine Travers, Joanne Tropea, Beibei Xiong, The Research Collaborative for Quality Care: Acute Care Panel, Alison Mudge, Jeffrey Rowland, Kwang Lim, The Research Collaborative for Quality Care: Dementia Care Panel, Elizabeth Beattie, Eddy Strivens, Paul Varghese

**Affiliations:** 1 Centre for Health Services Research, The University of Queensland, Building 33, Princess Alexandra Hospital, 34 Cornwall St, Brisbane, QLD 4102 Australia; 2 Department of Health and Life Sciences, University of Exeter, Exeter, UK; 3 Department of Clinical Epidemiology, Biostatistics and Health Services Research, Melbourne Health, The University of Melbourne and The Royal Melbourne Hospital, Melbourne, VIC Australia; 4 Department of Medicine, University of Melbourne, Parkville, VIC Australia; 5 Centre for Research Excellence in Patient Safety (CREPS), Monash University, Melbourne, VIC Australia; 6 Nutrition and Dietetics, School of Human Movement and Nutrition Sciences, The University of Queensland, Brisbane, QLD Australia; 7 School of Psychology, The University of Queensland, Brisbane, QLD Australia; 8 Mayne Academy of Psychiatry, Faculty of Medicine, The University of Queensland, Brisbane, QLD Australia; 9 Mental Health Service, Royal Brisbane and Women’s Hospital, Brisbane, QLD Australia; 10 The Warren Alpert Medical School, Brown University, Boston, MA USA; 11 Institute of Ageing Research, Boston, MA USA; 12 School of Health and Rehabilitation Sciences, The University of Queensland, Brisbane, QLD Australia; 13 Department of Aged Care, Royal Melbourne Hospital, Melbourne, VIC Australia

**Keywords:** Quality indicators, Acute care, Geriatrics, Quality of care

## Abstract

**Background:**

A range of strategies are available that can improve the outcomes of older persons particularly in relation to basic activities of daily living during and after an acute care (AC) episode. This paper outlines the original development of outcome-oriented quality indicators (QIs) in relation to common geriatric syndromes and function for the care of the frail aged hospitalized in acute general medical wards.

**Methods:**

**Design** QIs were developed using evidence from literature, expert opinion, field study data and a formal voting process. A systematic literature review of literature identified existing QIs (there were no outcome QIs) and evidence of interventions that improve older persons’ outcomes in AC. Preliminary indicators were developed by two expert panels following consideration of the evidence. After analysis of the data from field testing (indicator prevalence, variability across sites), panel meetings refined the QIs prior to a formal voting process.

**Setting:**

Data was collected in nine Australian general medical wards.

**Participants:**

Patients aged 70 years and over, consented within 24 h of admission to the AC ward.

**Measurements:**

The interRAI Acute Care – Comprehensive Geriatric Assessment (interRAI AC-CGA) was administered at admission and discharge; a daily risk assessment in hospital; 28-day phone follow-up and chart audit.

**Results:**

Ten outcome QIs were established which focused on common geriatric syndromes and function for the care of the frail aged hospitalized in acute general medical wards.

**Conclusion:**

Ten outcome QIs were developed. These QIs can be used to identify areas where specific action will lead to improvements in the quality of care delivered to older persons in hospital.

**Supplementary Information:**

The online version contains supplementary material available at 10.1186/s12877-024-04980-9.

## Background

Older persons who are hospitalized for acute illnesses are at risk of both losing independence and institutionalization [1, 2]. A range of strategies are available that can improve their outcomes at discharge, particularly in relation to the ability to perform basic activities of daily living and discharge to long term residential care [2]. The challenge is in identifying areas where limited time and resources should be focused to make appropriate changes.

Quality assurance activities can be complex and multifactorial. Prior literature tempers expectations between process and outcomes [3]. It can be challenging to record a link between changed processes and improved outcomes. Despite this, measuring patient outcomes and improving clinical practice to optimize patient outcomes in the long term are important. Quality indicators (QIs) (structure, process and outcome) are one way to measure quality of care [4]. QIs are commonly used in hospitals, but there are no published sets of outcome QIs for geriatric syndromes and function for the care of older persons in acute care (AC) prior to the interRAI AC QIs [5–7].

QI development is most effective when it includes expert opinion, a review of the scientific literature and field study data [8, 9]. Measuring quality of care based on patient outcomes has inherent appeal, but there are major concerns. There are attribution problems (difficulties measuring the aspect of quality precisely or exclusively) [1]; and problems with inaccurate trigger rates (triggering based on other random factors and resulting in inaccurate results). Depending on the location of the hospital, socio-economic and health demographic factors will also impact the bed-use and presentation profile. Any development of outcome QIs must take these factors into account [10] because a QI that measures a hospital’s performance is only meaningful when it measures factors that hospital can control, and includes mechanisms to adjust for factors that hospital cannot control.

The aim of this study was to develop outcome oriented QIs in relation to common geriatric syndromes and function for the care of the frail aged hospitalized in acute general medical wards. These QIs have since been adopted into an international assessment system for AC and a supplementary set created for use in a surgical setting. This paper outlines the original development project.

## Method

This project was carried out in three phases: Development of a preliminary QI set; field study; analysis and compilation of a definitive QI set. The research methodology for this project has been described in detail elsewhere [10]. A truncated description is provided here.

### Phase 1: Development of a preliminary QI set

#### Literature Summary

A scoping review of the scientific literature pertaining to adverse geriatric outcomes in acute hospitals was compiled. Publications with the highest levels of evidence in each domain of interest were identified together with existing guidelines and published QIs relevant to hospitalized older persons (with an interpretation of methodological quality and applicability).

### Expert panels

Given the scope of the project, two expert panels were required: panel one focused on generic outcomes for older persons (e.g., continence) [Acute Care Panel, Supplementary Box 1]; and panel two focused on cognitive health (e.g., the recognition of cognitive impairment) [Dementia Care Panel, Supplementary Box 2]. Expertise criteria for panel participation was established a priori. Experience included: allied health, consumer advocacy, dementia, general medicine, geriatric medicine, gerontic nursing, quality research methodology, and AC public hospital health care. Participating sites for data collection were recruited from two Australian states (Queensland, Victoria), and coordinators at each site participated in the expert panels. Based on matching site coordinator's experience with the a priori list, any remaining gaps were filled by invitation from the research team.

*Expert Panel Meetings.* The literature summaries were divided, based on relevance, between the two expert panels. Initially, each panel met for two days to review their respective summaries and conceptualise potential QIs. Panel members recommended QIs according to predefined criteria: being amenable to change, within the control of the hospital, had evidence to support a link between the actions of a hospital and the outcome, and which were clinically significant for an older person and therefore warranted attention. Each QI was defined (i.e. identifying the variables needed for calculating the numerator, denominator, inclusion, and exclusion criteria). The QIs were linked with variables from the interRAI AC – Comprehensive Geriatric Assessment (AC- CGA) tool [11] for data collection in the first instance, to score the QIs prior to evaluation in phase 3.

### Phase 2: Field Study

Patients aged 70 years and older admitted to nine Australian AC general medical hospitals, who were likely to stay in hospital for at least 48 h, were invited to participate. The data collection took an average of 7 months (range 4–10 months). Data collection period by hospital type (tertiary or regional) is shown on a radar chart in the Supplementary Fig. 1. Hospitals included five metropolitan tertiary teaching hospitals and four regional hospitals. Ethics approval was obtained from the relevant organisations.

### Data collection

Each hospital nominated one or two registered nurses with gerontic experience to work full-time on the project as data collectors during the study period. One full-time registered nurse with gerontic experience, trained in project data collection, was rostered each day per site for 7.5 h of data collection a day across five days each week (Monday to Friday) during the data collection period. All nurse assessors attended a three-day training program at the Centre for Health Services Research (CHSR) prior to commencing data collection. Training involved understanding the project protocol and minimising potential research bias, administration and scoring of the formal assessment tools, and the completion of the study recruitment database. A regular teleconference schedule was established for interaction between sites during the data collection period and protocols for data storage and transfer were put in place. All site research nurses were trained to administer the interRAI AC-CGA by the same experienced interRAI assessment trainer. Previous studies have shown very high levels of reliability on observed agreement for the interRAI AC-CGA [12, 13].

Demographic data was collected on patients who consented to participate in the study. The interRAI AC-CGA was administered within 24 h of admission to the ward for consenting patients, with daily follow-up and review at discharge. At 28 days, a follow-up telephone call to the patient’s or carer’s home was made to collect data on readmission and post-discharge change of living arrangement, this included the Mini-Mental State Examination 22-item telephone version (ALFI-MMSE 22-items) [14, 15]. The data collected at 28 days was part of demographic data and was not used in the calculation of any QIs. Verification of re-admission, including diagnostic codes, was obtained by State data custodians or the relevant hospital data custodian.

### Phase 3: Analysis and final consultation (with expert panels)

#### Statistical analysis

Data was entered into IBM SPSS Statistics v26 2019. General descriptive statistics (frequencies, standard deviations and means) were used to describe characteristics of enrolled participants and to compare between hospitals, using demographic data, and cognitive and functional scales derived from the interRAI AC-CGA.

Each QI was scored for the total sample, and for each individual hospital. A trigger rate (percentage) was calculated for each QI (the proportion of patients who triggered the indicator event (the numerator) divided by the total number of eligible patients (the denominator)).

The expert-panel-identified covariates were analysed for statistical relevance to the sample by presenting the QI trigger rates when the covariate was present and absent. This was followed by descriptive statistics (odds ratios with 95% confidence intervals, and p-values) to identify statistically significant factors for each QI. All covariate information was provided, noting that multiple risk adjustments cannot be applied simultaneously to this sample given the small sample size.

Using the RAND-UCLA consensus method [16] two rounds of voting for each panel were completed. Analysis of voting round data was completed after each round. For each QI the following were calculated using the panel members’ individual votes of 1–9: the 30th and 70th percentile, the Interpercentile Range Adjusted for Symmetry, the Interpercentile Range, Interpercentile Range Central Point, the Asymmetry Index, median and mean deviation from the median. This data were used to apply decision rules, defined a priori, to identify valid or invalid QIs [17]. Only votes from round 2 were utilised to determine the final QI set.

### Expert Panel

A report was prepared for each panel (there was no overlap of indicators between panels) with a description of the preliminary indicators suggested from Phases 1 and 2, feedback provided by the interRAI AC Network and the interRAI Instrument Committee, and any recommendations (Supplementary Fig. 2). Any new research published in the interim was also provided. Each panel held a second two-day workshop to scrutinise the preliminary QIs which had now been tested in the field. QIs were modified based on recommendations from the expert panel following interpretation of the field data, evidence from the literature, and expert opinion.

Risk Adjustment: Risk adjustment was considered for each QI separately at the final expert panel meetings. Risk adjustment was approved if there was agreement amongst the panel members that factors, outside the control of the hospital, would impact the QI’s outcome, and those factors couldn’t be dealt with by excluding patients from the denominator. Risk adjustment was not approved in situations where it was recognised that certain patient characteristics did pose extra challenges but this didn’t obviate the extra care that was required to ensure patients’ safety and well-being. For example, the Dementia Care panel recognized that patients with dementia were at higher risk of delirium in hospital, but this did not justify risk adjustment for the delirium QI. It was important that staff identify these patients and provide care to minimise the risk of delirium rather than accept that it was inevitable. For each QI, the prevalence of the outcome across all patients in hospital datasets, after exclusions, is the facility-level observed QI score. Risk adjustment was calculated, when the final set of outcome QIs was identified, using logistic regression models [10, 18].

### Voting

Each panel voted on an independent set of QIs which were combined to establish the final outcome QI set. Each member voted on a scale of 1–9 with reference to specific criteria. A second voting sheet was prepared individually for each panel member which showed the spread of panel votes for each QI and the personal vote of that panel member, along with the QI data analysis (Valid or not, etc.). A teleconference was held to discuss the round 1 results. Where the votes for a QI was wide-spread, it generally indicated a misunderstanding, a poorly constructed QI, or diverse opinion amongst the panel members. The teleconference enabled clarification and rectified any misunderstandings. Each panel member voted a second time to conclude the final outcome AC set.

## Results

Data from 643 patients at nine hospitals were analysed and used to calculate each individual QI. No patients withdrew during the in-hospital data collection. Characteristics of the included sample (Table 1) is provided.

**Table 1:** Characteristics of patients in the study

Variables	Total	Age < 75	Age 75–89	Age 90 +
	***N*= 643**	***N*= 128**	***N*= 442**	***N*= 73**
**Male**	297 (46%)	64 (50%)	205 (46%)	28 (38%)
**Residence at admission**				
Community Dwelling	559 (87%)	116 (91%)	388 (88%)	55 (75%)
Residential aged care	69 (11%)	6 (5%)	45 (10%)	18 (25%)
Other	15 (2%)	6 (5%)	9 (2%)	0 (0%)
**Admitted via Emergency**	560 (89%)	102 (81%)	388 (90%)	70 (97%)
**Hospitalised in last 30 days**	141 (22%)	32 (25%)	97 (22%)	12 (16%)
**Measure of ADL function prior to admission (ADL-SF, Range 0–16)**				
Mild	513 (80%)	115 (90%)	353 (80%)	45 (62%)
Limited to Maximal Impairment	67 (10%)	8 (6%)	40 (9%)	19 (26%)
Dependent	63 (10%)	5 (4%)	49 (11%)	9 (12%)
**Measure of Cognitive Function prior to admission (CPS, Range 0–6)**				
Intact or Borderline Intact	493 (77%)	117 (91%)	336 (77%)	40 (55%)
Mild/moderately severe impairment	121 (19%)	10 (8%)	82 (19%)	29 (40%)
Severe/very severe impairment	25 (4%)	1 (1%)	20 (5%)	4 (5%)
**Risk Assessment at Admission**				
Increased risk of ADL decline^a^ in hospital	52 (8%)	8 (6%)	36 (9%)	8 (12%)
No. of Comorbidities (Range 0–12) Median	7 (5–8)	6 (4–8)	7 (5–8)	6 (5–8)
Falls History Two or more falls in the last 30 days	78 (12%)	14 (11%)	45 (10%)	19 (26%)
**ICD Codes for Acute Admission**				
Circulatory system disease—Other heart disease	66 (10%)	10 (8%)	49 (11%)	7 (10%)
Respiratory disease—Influenza and pneumonia	46 (7%)	7 (6%)	30 (7%)	9 (12%)
Respiratory disease—Chronic lower respiratory diseases	70 (11%)	18 (14%)	47 (11%)	5 (7%)
Symptoms and signs not elsewhere classified	66 (10%)	12 (10%)	43 (10%)	11 (15%)
Injury, poisoning and other consequences of external causes	56 (9%)	3 (2%)	41 (9%)	12 (16%)
Other	336 (53%)	76 (60%)	231 (52%)	29 (40%)
**Length of Stay Acute Care**	6 (3–11)	6 (3–9)	7 (4–12)	6 (3–13)
**At least one adverse outcome**^**b**^** in hospital**	216 (34%)	27 (21%)	154 (35%)	35 (48%)
**Readmitted within 28 days of discharge**	125 (21%)	22 (18%)	91 (23%)	12 (18%)
**Died in 28 days post-discharge**				
No	584 (92%)	120 (94%)	397 (91%)	67 (92%)
Yes died within 28 days of discharge	27 (4%)	4 (3%)	21 (5%)	2 (3%)
Died at index hospitalisation	26 (4%)	3 (2%)	19 (4%)	4 (5%)
**Newly discharged from AC to RACF**	31 (5%)	2 (2%)	22 (5%)	7 (10%)

Data are presented as median (IQR) for continuous measures, and n (%) for categorical measures.

^a^Increased risk of activities of daily life (ADL) decline – ADL-SF score from premorbid between 2 and 16 or CPS score at admission between 2 and 5.

^b^Adverse outcome denotes the event of one or more of either fall, pressure area, decline in activities of daily living (assessed daily), delirium onset

### The Research Collaboration for Quality Care (RCQC) Outcome QIs for Older Persons in AC

**Fig. 1:**
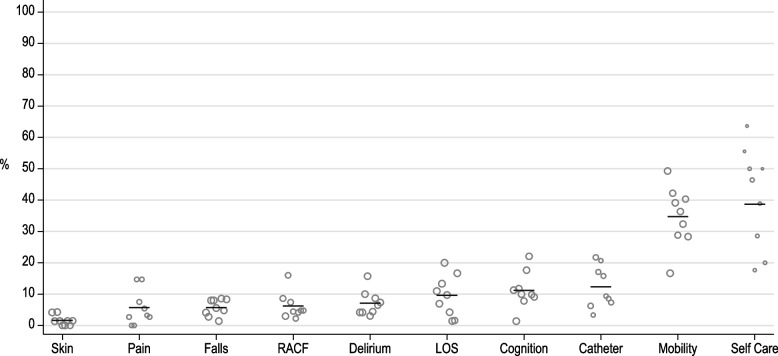
Field study data of outcome indicators for older adults, ordered by lowest to highest performing in terms of overall trigger rate**.** *Note: The size of the marker is scaled according to the denominator*

Ten outcome indicators were developed for the AC QIs for Older Persons. Table 2 describes each QI. Each QI has been classified according to the Institute of Medicine (IOM) framework, which is a useful way of considering the domains of care, and how this set of outcome QIs sit within those domains [19, 20]. The domains of care, as defined in the IOM framework are: safety, effectiveness, patient-centeredness, timeliness, efficiency, and equity. Figure 1 presents the QIs and displays the observed between site variation of scores within each QI. Table 3 provides raw summary data for each QI. In all cases, the QIs are interpreted by understanding that a lower score reflects a better outcome for the patient and the hospital.

**Table 2:** Research Collaboration for Quality Care - patient outcome quality indicators for older persons in acute care: definitions [20]

#	Short Title	Specifications	TIME POINT	IOM [20]	TYPE
1	Bladder Catheter	N: The proportion of female patients with a new urinary catheter on admission D: Female patients admitted E1: Presence of a urinary catheter premorbid E2: Male C1: Frequently or generally incontinent at Premorbid	Admission	Safe	Prevalence
2	Delirium^a^	N: The proportion of patients with delirium-indicating behaviours present at discharge D: Patients discharged E: Died prior to discharge C: None	Discharge	Safe	Prevalence
3	Cognition^a^†	N: The proportion of patients discharged with worse levels of cognitive function compared with premorbid levels D: Patients discharged E1: Died prior to discharge E2: Premorbid cognitive performance level such that no further decline could be identified C1: Resident of long term care C2: Impaired premorbid cognitive function C3: Surgery a part of acute episode	Discharge	Safe	Failure to Improve
4	Mobility^a^	N: Patients discharged with worse levels of mobility compared with pre-morbid levels D: Patients discharged E1: Died prior to discharge E2: Discharged to palliative care E3: Pre-morbid levels of mobility that couldn’t be rated for a further deterioration E4: Activity did not occur C1: Fall within last 90 days C2: Existing or new residential care facility living arrangements (pre-morbid or post-discharge) C3: Discharge to rehabilitation	Discharge	Effective	Failure to Improve
5	Self-Care^a^	N: The proportion of patients with pre-hospital decline who failed to return to pre-admission function (or better) by discharge D: Patients with a decline in function between premorbid and admission E1: Died prior to discharge E2: Discharged to rehabilitation E3: Discharged to palliative care E4: No decline in function at admission (when comparing admission with pre-morbid function) E5: Scale for QI could not be calculated C: None	Discharge	Effective	Failure to Improve
6	Pain	N: The proportion of patients with no pre-morbid pain who were discharged with unimproved pain when compared to reported pain at admission D: Patients discharged E1: Died prior to discharge E2: Premorbid report of pain C: None	Discharge	Effective	Failure to Improve
7	Skin Integrity	N: The proportion of patients with a new or worsening pressure injury at discharge compared with admission D: Patients discharged E: Died prior to discharge C1: Poor bed mobility premorbid C2: Nutritional issues C3: Prior pressure injury	Discharge	Effective	Failure to Improve
8	Falls	N: The proportion of patients who fell (at least once) during the hospital episode D: Patients admitted E: None C1: Low premorbid cognitive function C2: Fall within 90 days	Episode	Safe	Incidence
9	Prolonged Length of Stay	N: The proportion of patients with prolonged length of stay D: Patients discharged E: None C1: Discharge to rehabilitation	Episode	Efficient	Incidence
10	New Discharge to Residential Care	N: The proportion of community dwelling patients discharged to long term care D: Community dwelling patients discharged E1: Died prior to discharge E2: Referred to rehabilitation E3: Premorbid location which was not a community dwelling (private housing, independent living units, or boarding house) E4: Discharge to an alternative acute care hospital C1: Low premorbid cognitive function C2: Prior admission to hospital in the last 90 days C3: Premorbid problems with hygiene C4: Premorbid inability to manage finances	Discharge	Patient-Centered	Incidence

*N *Nominator,* D *Denominator,* C *Covariate,* E *Exclusion,* IOM *Institute of Medicine framework

^a^Derived using an interRAI Scale or specific variable; †Not in the interRAI Acute Care Quality Indicator Set

**Table 3:** Raw summary data for each quality indicator, including trigger rates (with p-values) for the whole sample, and each hospital

	**Total**	**1**	**2**	**3**	**4**	**5**	**6**	**7**	**8**	**9**	***p***-value
	***N*= 643**	***n*= 73**	***n*= 72**	***n*= 75**	***n*= 75**	***n*= 72**	***n*= 71**	***n*= 70**	***n*= 63**	***n*= 72**	
Bladder Catheter	42/340 (12%)	3/48 (6%)	1/30 (3%)	10/46 (22%)	7/41 (17%)	6/29 (21%)	6/38 (16%)	3/32 (9%)	3/35 (9%)	3/41 (7%)	0.13
Delirium	44/617 (7%)	3/72 (4%)	3/72 (4%)	7/70 (10%)	11/70 (16%)	2/66 (3%)	3/68 (4%)	6/69 (9%)	4/62 (6%)	5/68 (7%)	0.10
Cognition	68/608 (11%)	8/71 (11%)	1/72 (1%)	8/68 (12%)	7/70 (10%)	5/64 (8%)	12/68 (18%)	15/68 (22%)	6/61 (10%)	6/66 (9%)	0.014
Mobility	207/596 (35%)	34/69 (49%)	12/72 (17%)	27/64 (42%)	27/69 (39%)	17/59 (29%)	24/66 (36%)	22/68 (32%)	25/62 (40%)	19/67 (28%)	0.005
Self-Care	58/150 (39%)	5/9 (56%)	7/11 (64%)	9/18 (50%)	13/28 (46%)	3/17 (18%)	6/21 (29%)	7/18 (39%)	4/8 (50%)	4/20 (20%)	0.11
Pain	18/315 (6%)	1/37 (3%)	0/33 (0%)	0/32 (0%)	5/34 (15%)	3/40 (8%)	5/34 (15%)	2/37 (5%)	1/31 (3%)	1/37 (3%)	0.043
Skin Integrity	10/617 (2%)	3/72 (4%)	1/72 (1%)	3/70 (4%)	1/70 (1%)	0/66 (0%)	0/68 (0%)	1/69 (1%)	0/62 (0%)	1/68 (1%)	0.31
Falls	37/643 (6%)	3/73 (4%)	2/72 (3%)	6/75 (8%)	6/75 (8%)	4/72 (6%)	1/71 (1%)	6/70 (9%)	3/63 (5%)	6/72 (8%)	0.49
Prolonged Length of Stay	62/643 (10%)	8/73 (11%)	5/72 (7%)	10/75 (13%)	15/75 (20%)	7/72 (10%)	3/71 (4%)	1/70 (1%)	1/63 (2%)	12/72 (17%)	< 0.001
New Discharge to Residential Care	28/451 (6%)	5/58 (9%)	2/67 (3%)	8/50 (16%)	4/54 (7%)	2/45 (4%)	1/45 (2%)	2/48 (4%)	2/42 (5%)	2/42 (5%)	0.14

*Data are presented as n/total (%). P-value from Chi-square test*

^*^Statistically significant (p < *0.05)*

### RCQC Outcome QIs for older persons in AC:

1. **The proportion of female patients with a new urinary catheter on admission.** Functional decline guidelines recommend careful assessment of the need for an indwelling urinary catheter prior to use given the evidence of a causal link between indwelling catheter use and urinary tract infections [21, 22]. The focus of this QI is on female patients as it was felt that clinically there was less justification for using catheters in women than men and that this better represented quality of care (face validity) than a QI for all patients.
2. **The proportion of patients with delirium-indicating behaviors present at discharge.** There is evidence for delirium reducing interventions in AC. Australian functional decline guidelines (expert opinion levels of evidence) recommend consideration of transitional care needs and community-based strategies for people discharged from hospital and residential care to take into account delirium issues [21, 22]. This QI considers the potential risk of discharging older people from hospital if they have delirium, without firstly being aware that delirium is present, and second using clinical judgement to decide if discharge is the best course of action. The panel recognises that in some cases, with careful discharge planning, the best choice for a patient with delirium may be discharge.
3. **The proportion of patients discharged with worse levels of cognitive function compared with premorbid levels. **Declining Mini-Mental State Examination scores have been shown to predict mortality [23]. Interventions can change the outcome and assist in maintaining cognitive functions [24]. There is variation in recoverable cognitive dysfunction amongst older patients in AC [25]. This QI was included by the panel because of the impact of cognitive decline, over and above that of delirium, for older patients.
4. **The proportion of patients discharged with worse mobility levels compared with pre-morbid levels.** Levels of mobility, self-care needs and cognitive and behavioral status are factors often associated with needing additional ‘non-acute’ care while in hospital. This extra care is more commonly needed by older people who have issues with mobility when in hospital. Addressing support for mobility (and other self-care needs) in hospital impacts future care needs for older patients (i.e. after the hospital episode) [26]. The panel acknowledge the high level evidence in support of this QI, which indicates the use of multidisciplinary interventions (which include a component of exercise) to reduce hospital length of stay, increase proportion of patients discharged home and reduce costs of stay for older acute hospitalized inpatients [21, 22].
5. **The proportion of patients with pre-hospital decline who failed to return to preadmission function (or better) by discharge.** There is a significant body of evidence that shows older people decline in function by the time of discharge from AC, and that this negatively impacts quality of life of hospitalized older people [27]. Recommendations of the Australian Functional Decline Guidelines, based on expert opinion, include individualized care plans which encourage appropriate incidental activity throughout the day and minimize bed rest, and recommendations to assess and modify the environment to encourage independence and mobility [21, 22].
6. **The proportion of patients with no pre-morbid pain who reported both pain at admission and unimproved pain at discharge.** There is evidence that improved strategies to manage pain (i.e. quality improvement collaborative program) improve processes of care (such as improved use of pain assessment tools), but only limited evidence of links to patient outcomes (i.e. sufficient pain treatment for patients) [28, 29].
7. **The proportion of patients with a new or worsening pressure ulcer at discharge when compared with admission. **A review of studies reporting the implementation of pressure ulcer reduction strategies (*n* = 16) reported a positive reduction in the outcome pressure ulcer incidence [30]. There is strong evidence to link specific practices in AC to reducing the risk of pressure ulcers (in patients with high risk) [21, 22]. The panel recognized that the incidence of pressure ulcers was small in this study but that the clinical impact was significant and therefore it was included as an indicator of quality of care.
8. **The proportion of patients who fell (at least once) during the hospital episode. **Some falls are preventable while some are not preventable [31]. There is sound evidence to support the use of multifaceted approaches to reduce the falls rate in hospital settings. There is some evidence to support the use of specialist geriatric wards to reduce falls incidence and fall-related injuries among post-operative hip fracture patients [32, 33]. The panel recognized that the incidence of falls was small in this study but that the clinical impact for older persons was significant and therefore it was included as an indicator of quality of care.
9. **The proportion of patients with prolonged length of stay.** Generally, injured older adults (> = 65 years) have an average length of hospital stay, including admissions to ICU, exceeding 10 days. A retrospective study of the characteristics and outcomes of injured older adults after hospital admission (N = 6,069) from the Queensland Trauma Registry identified that the average length of stay (including patients in ICU) was eight days (interquartile range 5–15), with 33.8% of cases with a major injury developing a complication [34]. There is evidence to indicate that the risk of iatrogenic complications is higher if an older person is hospitalized for longer than one week. These complications are frequently preventable and are often associated with a rapid decline in muscle strength, aerobic capacity and pulmonary ventilation that older patients experience [35]. For the majority of older patients, ‘waiting for placement’ is not a contributor to their length of stay. Most older people are discharged to their usual place of residence [36].
10. **The proportion of community-dwelling patients discharged to long-term care.** There is good evidence to support the use of multidisciplinary interventions that include a component of exercise to reduce in-hospital length of stay, increase the proportion of patients discharge directly home and reduce costs of stay for older acute hospitalised inpatients [21, 22]. A review focused on patients regaining functional independence in the acute setting following hip fracture concluded that patients who stayed longer in the acute setting, received physical therapy more than once a day had improved odds of regaining independence in bed mobility, transfers and ambulation. Patients who regained independence along with use of physical therapy services had improved odds of being discharged directly to the home from the acute setting [37].

No covariates for risk adjustment were recommended for three QIs: Delirium, Self Care, and Pain. The remaining QIs each had covariates recommended for risk adjustment (Table 4). The initial step for industry uptake has been swift. The final set of QIs were presented to the interRAI Instrument Committee. The *RCQC Patient Outcome QIs for Older Persons in AC* were formally endorsed by interRAI and adopted (except for the cognition indicator) for use with the interRAI AC suite [7, 11] with modification recommended for the surgical context [38]. Software vendors who provide electronic versions of the interRAI AC now also include algorithms to run the AC outcome indictors derived from the data collected by staff when completing care assessments.

**Table 4:** Recommended covariates and examination of factors associated with risk adjustment

QI Short Title	Recommended Covariate	Unadjusted QI Rate			Effect of covariate on QI Trigger rate		Covariate adjusted QI trigger rate (%)									
		Overall n/D (%)	Covariate present n/D (%)	Covariate absent n/D (%)	Odds Ratio (95 CI)	p-value	Range across sites	1	2	3	4	5	6	7	8	9
**Bladder Catheter**	C1: Incontinent Premorbid; Female	42/340 (12%)	11/64 (17%)	31/276 (11%)	1.6 (0.8, 3.5)	.21	3—21	7	3	20	17	21	16	9	9	7
**Cognition**	C1: Resident of long-term care	68/608 (11%)	19/77 (25%)	49/530 (9%)	3.2 (1.8, 5.8)	< .001	2—22	11	2	11	10	9	17	22	9	8
	C2: Impaired premorbid cognitive function		3/20 (15%)	65/588 (11%)	1.4 (0.4, 5.0)	.48	1—22	11	1	12	10	8	18	22	10	9
	C3: Surgery a part of acute episode		0/12 (0%)	67/587 (11%)	n/a	.38	2—22	11	2	12	10	8	18	22	10	9
**Mobility**	C1: Fall within last 90 days	207/596 (35%)	109/233 (47%)	98/363 (27%)	2.4 (1.7, 3.4)	< .0001	20—52	52	20	40	38	27	36	31	41	27
	C2: Existing or new RACF living arrangements		41/84 (49%)	166/512 (32%)	2.0 (1.2, 3.2)	< .01	18—49	49	18	40	39	30	36	32	41	28
	C3: Discharge to rehabilitation		37/50 (74%)	170/546 (31%)	6.3 (3.3, 12.1)	< .0001	18—52	52	18	42	43	31	34	29	40	25
**Skin Integrity**	C1: Poor Bed mobility premorbid	10/617 (2%)	4/48 (8%)	6/569 (1%)	8.5 (2.3, 31.4)	< .01	0—4	4	2	4	1	0	0	2	0	1
	C2: Nutritional issues		1/105 (1%)	9/505 (2%)	0.5 (0.1, 4.2)	1	0—4	4	1	4	1	0	0	1	0	1
	C3: Prior pressure injury		2/42 (5%)	8/569 (1%)	3.5 (0.7, 17.1)	.15	0—4	4	1	4	1	0	0	2	0	2
**Falls**	C1: Low premorbid cognitive function	37/643 (6%)	3/25 (12%)	34/611 (6%)	2.3 (0.7, 8.1)	.17	1—9	4	3	8	8	6	1	9	5	8
	C2: Fall within 90 days		20/249 (8%)	17/394 (4%)	1.9 (1.0, 3.8)	.056	1—8	4	3	8	8	5	1	8	5	8
**Prolonged Length of Stay**	C1: Discharged to rehabilitation	62/643 (10%)	9/51 (18%)	53/592 (9%)	2.2 (1.0, 4.7)	.078	1—21	11	7	13	21	10	4	1	2	16
**New Discharge to Residential Care**	C1: Low premorbid cognitive function	28/451 (6%)	3/7 (43%)	25/440 (6%)	12.4 (2.6, 58.7)	< .01	2—14	9	3	14	7	5	2	4	5	4
	C2: Prior admission to hospital in the last 90 days		14/183 (8%)	14/268 (5%)	1.5 (0.7, 3.2)	.32	2—16	9	3	16	7	5	2	4	5	5
	C3: Premorbid problems with hygiene		10/56 (18%)	18/395 (5%)	4.6 (2.0, 10.5)	< .001	2—14	7	3	14	7	5	2	4	6	5
	C4: Premorbid inability to manage finances		22/174 (13%)	6/277 (2%)	6.5 (2.6, 16.5)	< .0001	3—16	8	4	16	7	4	3	3	7	4

*C*are assessment and planning targeting all patients at all times have no covariate recommendations: Delirium, Self-Care, and Pain. *QI* quality indicator

*Statistically significant (*p* < 0.05)

## Discussion

The aim of this project was to develop outcome-oriented QIs in relation to common geriatric syndromes and function for the care of the frail aged hospitalized in acute general medical wards derived from the interRAI AC-CGA assessment tool clinical data items. The interRAI assessments were selected because they are implemented as care focused sources of data collection. From that care assessment data, a sub-set of items are used to score the QIs, but it is assumed that the assessment is collected in full initially by care staff for the primary purpose of delivering care to a patient in a health service. There is no additional burden of collecting data for administrative or quality care purposes. Many of the QIs can be scored without the interRAI AC-CGA, or they can be scored using a measure the facility currently uses if only internal benchmarking is occurring. QIs have been clearly marked in Tables 2 and Supplementary Table 1 to indicate where this applies.

Ten outcome QIs have been developed, with nine formally endorsed by interRAI for use in the interRAI AC suite (except for the cognition indicator as it was decided delirium was the primary quality issue relevant in AC at this time). The interRAI AC suite is a set of tools, primarily used by nurses at a ward level for risk assessment, to guide clinical care planning and preparation for specialist consultation. The QIs were developed to support improvements in clinical care at the ward level. They can be used to benchmark existing levels of care, and measure change after the implementation of interventions. Future work in quality improvement and outcome QIs will focus on understanding the extent to which staff self-direct the modification of their clinical activities when they are aware of the QI scoring.

When the study was carried out originally the primary data collection tool was the interRAI AC-CGA (a longer assessment). There is now a shorter nursing assessment, a 15-min interRAI AC, and supplementary surgical QIs have been developed. The Supplementary Table 1 illustrates how the QIs can be scored using administrative or clinical data, or interRAI data (i.e. Some QIs can be scored without the interRAI AC as they are straightforward prevalence or incidence indicators). These are listed in the Supplementary Table 1 and indicated with an * including bladder catheter, pain, skin integrity, falls, prolonged stay, institutional placement. At present, some QIs are scored using algorithms calculated from assessment data in the interRAI AC-CGA and the interRAI AC (delirium, cognition, self-care, mobility). Detailed information regarding the scoring is available in the interRAI clinical and applications manual for the interRAI AC [39] which is sourced at the interRAI website (www.interRAI.com). For example, QIs scored using scales inherent in the interRAI AC, and if the QI was to be implemented and scored using a different system (such as a cognitive tool, rather than the interRAI cognitive CPS scale), the benchmarks in this paper would not be comparable. The QIs scored using interRAI scales or specific items are marked as such in Table 2 and the Supplementary Table 1: these are the Delirium, Cognition, Mobility and Self Care QIs. For organisations not using the interRAI assessment to score these QIs, it is possible to use validated assessments as measures for internal purposes (for example, when scoring the delirium QI) but this will not enable immediate comparison for external benchmarking purposes as different measures have been used, nor can the data in this paper be used as a baseline.

We acknowledge the limitation of this project, that it was carried out in general medical wards at nine Australian hospitals in two States. We recognize that geriatric specific wards would have the potential to score differently as it is possible that the specific training and expertise that staff have in these units would change the outcomes for older persons (possibly improved outcomes; lower scores). The application of these QIs in general medical wards internationally may also generate different results, despite this, generalizability is not impacted as the criteria for quality of care is the same around the world. We were also limited in the information we could collect regarding participating patients and did not have consistency across all sites. This information would have increased our understanding of the generalisability of the findings and will be a strong focus for future work. Second, this paper describes the development of a set of outcome QIs. Further research to show the extent to which these indicators respond to interventions designed to improve patient outcomes is needed to validate their use in benchmarking quality improvement activities. The current set of indicators have been selected based on face validity in relation to responsiveness (i.e. there is published evidence that interventions can change the outcome in the area of interest). The interRAI AC-CGA has been used as the minimum data set for the development of the QIs (and they can also be scored on the companion interRAI AC). A strength in this approach is that interRAI assessments are an accepted international standard for minimum datasets and in many countries the assessments are mandated as part of health care data collection in a variety of health care settings. This creates an opportunity for these QIs to be utilised for international benchmarking in acute care in the future. Conversely, uptake maybe hindered by organisations which choose not to access licensed standardised assessment tools for their data collection and patient assessment. For those organisations, sufficient information is in the public domain for internal benchmarking activities utilising these QIs without the interRAI AC assessment but this limits the potential for international benchmarking.

Prior to this study, there were no published outcome indicators for older persons in AC. This work was a first step towards supporting improvement in the care of older persons in an acute setting. In their current format, these QIs can be applied in an acute hospital ward and the results can be compared with the field study data listed here as a benchmark. Poor scores on particular QIs can help to focus quality assurance time and resources to improving care where it is needed most.

## Conclusions

Two expert panels were formed to review the scientific literature and interpret field study data to develop a set of outcome QIs. Ten outcome QIs were developed. These QIs can be used to identify areas where specific action will lead to improvements in the quality of care delivered to older persons in hospital.

### Supplementary Information

**Supplementary Material 1.**

## Data Availability

The datasets generated and/or analysed during the current study are not publicly available due to privacy or ethical restrictions but are available from the corresponding author on reasonable request.

## Abbreviations

QI: Quality Indicator
AC: Acute Care
interRAI AC-CGA: interRAI Acute Care - Comprehensive Geriatric Assessment
RCQC: Research Collaboration for Quality Care

## References

[1] Anell A, Glenngård AH (2014). The use of outcome and process indicators to incentivize integrated care for frail older people: a case study of primary care services in Sweden. Int J Integr Care.

[2] Landefeld CS, Palmer RM, Kresevic DM, Fortinsky RH, Kowal J (1995). A randomized trial of care in a hospital medical unit especially designed to improve the functional outcomes of acutely ill older patients. N Engl J Med.

[3] Schneider EC (2014). Hospital quality management: a shape-shifting cornerstone in the foundation for high-quality health care. International Journal For Quality In Health Care: Journal Of The International Society For Quality In Health Care / Isqua.

[4] Donabedian A (1988). The quality of care: How can it be assessed?. JAMA.

[5] Ahmed N, Taylor K, McDaniel Y, Dyer CB (2012). The role of an Acute Care for the Elderly unit in achieving hospital quality indicators while caring for frail hospitalized elders. Popul Health Manag.

[6] Katterl R, Anikeeva O, Butler C, Brown L, Smith B, Bywood P. Potentially avoidable hospitalisations in Australia: Causes for hospitalisations and primary health care interventions. PHC RIS Policy Issue Review. Adelaide: Primary Health Care Research & Information Service; 2012. https://core.ac.uk/reader/14947500.

[7] Gray LC, Beattie E, Boscart VM, Henderson A, Hornby-Turner YC, Hubbard RE, Wood S, Peel NM (2018). Development and testing of the interRAI acute care: a standardized assessment administered by nurses for patients admitted to acute care. Health Services Insights.

[8] De Koning J, Burgers JS, Klazinga N. Appraisal of Indicators through Research and Evaluation (English version based on original Dutch version 2.0). Amsterdam: University of Amsterdam; 2007.

[9] Burkett E, Martin-Khan MG, Gray LC (2017). Quality indicators in the care of older persons in the emergency department: A systematic review of the literature. Australas J Ageing.

[10] Brand C, Martin-Khan M, Wright O, Jones R, Morris J, Travers C, Tropea J, Gray L (2011). Development of quality indicators for monitoring outcomes of frail elderly hospitalised in acute care health settings: Study Protocol. BMC Health Services Research.

[11] Gray L, Arino-Blasco S, Berg K, Fries B, Heckman G, Jonsson P, Kergoat M-J, Morris J, Peel N, Sinha S (2017). interRAI Acute Care for Comprehensive Geriatric Assessment (AC-CGA) Form and User’s Manual Version 9.3.

[12] Wellens NI, Van Lancker A, Flamaing J, Gray L, Moons P, Verbeke G, Boonen S, Milisen K (2012). Interrater reliability of the interRAI Acute Care (interRAI AC). Arch Gerontol Geriatr.

[13] Wellens NI, Deschodt M, Boonen S, Flamaing J, Gray L, Moons P, Milisen K (2011). Validity of the interRAI Acute Care based on test content: a multi-center study. Aging Clin Exp Res.

[14] Roccaforte WH, Burke WJ, Bayer BL, Wengel SP (1992). Validation of a Telephone Version of the Mini-Mental State Examination. J Am Geriatr Soc.

[15] Kennedy RE, Williams CP, Sawyer P, Allman RM, Crowe M (2014). Comparison of in-person and telephone administration of the Mini-Mental State Examination in the University of Alabama at Birmingham Study of Aging. J Am Geriatr Soc.

[16] Fitch K, Bernstein S, Aguilar M, Burnand B, LaCalle J, Lazaro P, van het Loo M, McDonnell J, Vader J, Kahan J (2001). The RAND/UCLA appropriateness method user's manual.

[17] Basger BJ, Chen TF, Moles RJ (2012). Validation of prescribing appropriateness criteria for older Australians using the RAND/UCLA appropriateness method. BMJ Open.

[18] Abt Associates Inc. National nursing home quality measures: User’s manual. Cambridge, MA: Abt Associates Inc.; 2004. https://www.cms.gov/Medicare/Quality-Initiatives-Patient-Assessment-Instruments/NursingHomeQualityInits/downloads/nhqiqmusersmanual.pdf.

[19] Institute of Medicine (US) Committee on Quality of Health Care in America. Crossing the Quality Chasm: a New Health System for the 21st Century. Washington, DC: National Academies Press (US); 2001. 10.17226/10027. 25057539

[20] Romano P, Hussey P, Ritley D. Selecting Quality and Resource Use Measures: A Decision Guide for Community Quality Collaboratives. Rockville, MD, USA: US Department of Health and Human Services, Agency for Healthcare Research and Quality; 2010. https://www.ahrq.gov/sites/default/files/publications/files/perfmeas.pdf.

[21] Clinical Epidemiology and Health Service Evaluation Unit, Melbourne Health. Best practice approaches to minimise functional decline in the older person across the acute, sub-acute and residential aged care sectors. Melbourne, VIC: Victorian Government Department of Human Services; 2004. https://www.vgls.vic.gov.au/client/en_AU/search/asset/1160801/0.

[22] Clinical Epidemiology and Health Service Evaluation Unit, Melbourne Health. Best practice approaches to minimise functional decline in the older person across the acute, sub-acute and residential aged care sectors: Update. Melbourne, VIC: Victorian Government Department of Human Services; 2007. https://www.vgls.vic.gov.au/client/en_AU/search/asset/1161133/0.

[23] Lavery LL, Dodge HH, Snitz B, Ganguli M (2009). Cognitive decline and mortality in community-based cohort: the Monongahela valley independent elders survey. J Am Geriatr Soc.

[24] Inouye SK, Bogardus ST, Baker DI, Leo-Summers L, Cooney LM (2000). The hospital elder life program: a model of care to prevent cognitive and functional decline in older hospitalized patients. J Am Geriatr Soc.

[25] Chen CC-H, Chiu M-J, Chen S-P, Cheng C-M, Huang G-H (2011). Patterns of cognitive change in elderly patients during and 6 months after hospitalisation: A prospective cohort study. Int J Nurs Stud.

[26] To T, Davies O, Sincock J, Whitehead C (2010). Multidisciplinary care needs in an Australian tertiary teaching hospital. Aust Health Rev.

[27] Mudge A, O’Rouke P, Denaro C (2010). Timing and Risk Factors for Functional Changes Associated with Medical Hospitalisation in Older Patients. J Gerontol A Biol Sci Med Sci.

[28] Morrison R, Meier D, Fischberg D, Moore C, Degenholtz H, Litke A, Maroney-Galin C, Siu A (2006). Improving the management of pain in hospitalised adults. Arch Intern Med.

[29] Haller G, Agoritsas T, Luthy C, Piguet V, Griesser A, Perneger T (2011). Collaborative Quality Improvement to Manage Pain in Acute Care Hospitals. Pain Med.

[30] Soban L, Hempel S, Munjas B, Miles J, Rubenstein L (2011). Preventing Pressure ulcers in hospitals: a systematic review of nurse-focused quality improvement interventions. The Joint Commission Journal on quality and Patient Safetythe Joint Commission Journal on quality and Patient Safety.

[31] Australian Commission on Safety and Quality in Health Care (ACSQHC). Preventing falls and harm from falls in older people: Best practice guidelines for Australian hospitals. Sydney, NSW: Commonwealth of Australia ACSQHC. 2009. https://www.safetyandquality.gov.au/publications-and-resources/resource-library/preventing-falls-and-harm-falls-older-people-best-practice-guidelines-australian-hospitals.

[32] Australian Health Ministers’ Advisory Council (AHMAC). Best Practice Approaches to Minimise Functional Decline in the Older Person across the Acute, Sub-Acute and Residential Aged Care Settings. Melboure: Victorian Government Department of Human Services; 2004.

[33] Cameron I, Murray G, Gillespie L, Robertson M, Hill K, Cumming R, Kerse N (2010). Interventions for preventing falls in older people in nursing care facilities and hospitals. Cochrane Database Syst Rev..

[34] Aitken L, Burmeister E, Lang J, Chaboyer W, Richmond T (2010). Characteristics and outcomes of injured older adults after hospital admission. J Am Geriatr Soc.

[35] Creditor M (1993). Hazards of hospitalization of the elderly. Ann Intern Med.

[36] Gray L, Dakin L (2011). Australian and New Zealand Society for Geriatric Medicine Position statement-older persons in acute hospitals awaiting transfer to a residential aged care facility. Australas J Ageing.

[37] Guccione A, Fagerson T, Anderson J (1996). Regaining functional independence in the acute care setting following hip fracture. Phys Ther.

[38] Wood T, Chatfield M, Gray L, Peel N, Freeman S, Martin-Khan M (2022). Examining the adaptability and validity of interRAI acute care quality indicators in a surgical context. SAGE Open Medicine.

[39] Gray L, Arino-Blasco S, Berg K, Bula C, Gambassi G, Heckman G, Jonsson P, Kergoat M, Leff B, Martin-Khan M (2013). interRAI clinical and management applications manual: for use with the interRAI Acute Care Assessment Instrument Version 9.1.

