# Supplementary material for: Patient outcome quality indicators for older persons in acute care: original development data using interRAI AC-CGA
Source: BMC Geriatr. 2024 Jun 17;24:527. doi: 10.1186/s12877-024-04980-9 (PMC11184687; doi:10.1186/s12877-024-04980-9)
Supplement: Supplementary file 1 — Supplementary Material 1. [file 12877_2024_4980_MOESM1_ESM.docx]

**Supplementary File**

**Supplementary Box 1*.* Expertise of the RCQC Older Persons Acute Care Panel**

*The expert panel met for three days on two occasions and deliberated on the outcome indicators for older persons in acute care, and provided considerable time to review and voting for the final indicator set. The expertise of the RCQC Older Persons Acute Care Panel are:*

Geriatrician, Public Research Group of Eight University, QLD

Dietician, Public Research Group of Eight University, QLD

General physician, Metropolitan Tertiary Hospital, QLD

Geriatrician/Site Coordinator, Metropolitan Tertiary Hospital, QLD

Geriatrician/Site Coordinator, Metropolitan Tertiary Hospital, VIC

Rheumatologist and Quality Expert, Public Research Group of Eight University; Site coordinator, Metropolitan Tertiary Hospital, VIC

Academic Physiotherapist (Falls), Public Research Group of Eight University, QLD

Geriatrician/Site Coordinator, Metropolitan Tertiary Hospital, VIC

Social Worker, State Healthcare, QLD

Site Coordinator, Regional Hospital, QLD

Clinical Nurse, Regional Hospital, QLD

Geriatrician, Metropolitan Tertiary Hospital, VIC

Note: Some consortia members, who were available to approve publication, are included as co-authors in this publication. However, the expertise of all consortia members is listed here for completeness and clarity.

**Supplementary Box 2. Expertise of the RCQC –Older Persons Dementia Care Panel**

*The expert panel met for three days on two occasions and deliberated on the outcome indicators for older persons in acute care with specific reference to cognitive impairment, and provided considerable time to review and voting for the final indicator set. The expertise of the RCQC Older Persons Dementia Care Panel are:*

Emeritus Professor (Aged and Dementia Care), Public Research University, QLD

Psychogeriatrician, Public Research Group of Eight University, QLD; Site coordinator, Metropolitan Tertiary Hospital, QLD

Geriatrician, Public Research Group of Eight University, QLD

Psychologist, Public Research Group of Eight University, QLD

Geriatrician, Public Research University, QLD; Site coordinator, Regional Tertiary Hospital, QLD

Geriatrician/Site coordinator, Metropolitan Tertiary Hospital, QLD

Geriatrician, Metropolitan Tertiary Hospital, QLD

Rheumatologist and Quality Expert, Public Research Group of Eight University, VIC; Site coordinator, Metropolitan Tertiary Hospital, VIC

Consumer Representative, Alzheimer’s Australia, QLD

Psychogeriatrican, State Healthcare, QLD

Consumer Representative, Alzheimer’s Australia, QLD

Geriatrician, Metropolitan Tertiary Hospital, VIC

Clinical Neuropsychologist, State Healthcare, QLD

Note: Some consortia members, who were available to approve publication, are included as co-authors in this publication. However, the expertise of all consortia members is listed here for completeness and clarity.

**Supplementary Figure 1. Data collection period by hospital type (Tertiary or Regional)**

**Supplementary Figure 2. Process for panels to reach 10 outcome quality indicators (QIs)**


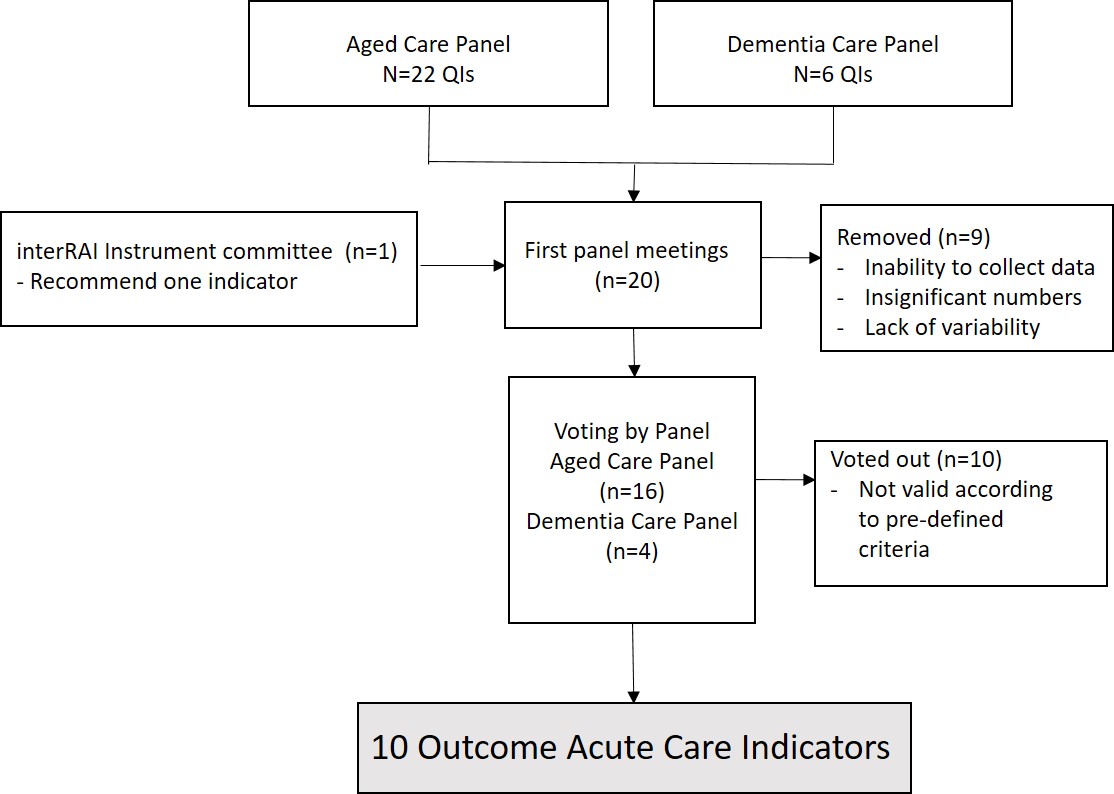


**Supplementary Table 1. Quality Indicators (QI) derived from administrative or clinical data, and from interRAI AC or AC-CGA [1]**

| **#** | **Short Title** | **General Medical Older Adult AC QI** | **Older Adult Surgical AC QI [2]** | **QI focus variable** | **interRAI scale or Item** | **interRAI AC [3]** | **interRAI AC-CGA [4]** |
| --- | --- | --- | --- | --- | --- | --- | --- |
| 1 | Bladder Catheter | The proportion of female patients with a new urinary catheter on admission | **[Elective and Non-elective]**  The proportion of female patients with a new urinary catheter present at 48-hours | Urinary Catheter | Indwelling urinary catheter | X | X |
| 2 | Cognitive Health (Delirium*) | The proportion of patients with delirium-indicating behaviours present at discharge^#^ | No change | Delirium | Delirium Scale [5]   - Easily distracted - Disorganised Speech - Mental function varies - Acute Change in mental status | X | X |
| 3 | Cognition*† | The proportion of patients discharged with worse levels of cognitive function compared with premorbid levels^#^ | N/A | Cognitive impairment | Cognitive Performance Scale [6]   - Cognitive skills for daily decision making - Memory/Recall ability: Short term memory (5 minutes); Procedural memory - Making self understood - ADL self-performance (feeding oneself) | X | X |
| 4 | Mobility* | Patients discharged with worse levels of mobility compared with pre-morbid levels | **[Elective]** *No change*  **[Non-elective]** The proportion of non-elective surgical patients with worse levels of walking between surgical baseline and discharge who also did not improve postoperative to discharge | Walking | Walking, or means of moving between a and b | X | X |
| 5 | Self-Care* | The proportion of patients with pre-hospital decline who failed to return to pre-admission function (or better) by discharge | **[Elective]** *No change*  **[Non-elective]** The proportion of non-elective surgical patients with a decline in function between surgical baseline and 48-hours postoperative who fail to return to surgical baseline function (or better) by discharge | Activities of Daily Living self-performance | ADL Scale [7]   - Personal hygiene - Walking - Toilet Use - Eating | X | X |
| 6 | Pain | The proportion of patients with no pre-morbid pain who were discharged with unimproved pain when compared to reported pain at admission | **[Elective and Non-elective]**  The proportion of patients with no premorbid pain who were discharged with unimproved pain when compared to reported postoperative pain at 48-hours | Pain | Pain intensity | X | X |
| 7 | Skin Integrity | The proportion of patients with a new or worsening pressure injury at discharge compared with admission | No change | Pressure injury | Most severe pressure injury | X | X |
| 8 | Falls | The proportion of patients who fell (at least once) during the hospital episode | No change | Fall | Fall | X | X |
| 9 | Prolonged Stay | The proportion of patients with prolonged length of stay | No change | Length of Stay | Length of Stay | X | X |
| 10 | Institutional placement | The proportion of community dwelling patients discharged to long term care | No change | Place of residence | Place of residence | X | X |

**Derived using an interRAI Scale or specific variable; †Not in the interRAI Acute Care Quality Indicator Set*

References for Supplementary Table 1:

1. Gray L, Arino-Blasco S, Berg K, Fries B, Heckman G, Jonsson P, Kergoat M, Morris J, Peel N, Sinha S: **interRAI Acute Care for Comprehensive Geriatric Assessment (AC-CGA) form and user’s manual**. 2017.

2. Wood T, Chatfield M, Gray L, Peel N, Freeman S, Martin-Khan M: **Examining the adaptability and validity of interRAI acute care quality indicators in a surgical context**. *SAGE Open Medicine* 2022, **10**:20503121221103221.

3. Gray LC, Beattie E, Boscart VM, Henderson A, Hornby-Turner YC, Hubbard RE, Wood S, Peel NM: **Development and testing of the interRAI acute care: a standardized assessment administered by nurses for patients admitted to acute care**. *Health Services Insights* 2018, **11**:1178632918818836.

4. Gray L, Arino-Blasco S, Berg K, Fries B, Heckman G, Jonsson P, Kergoat M-J, Morris J, Peel N, Sinha S *et al*: **interRAI Acute Care for Comprehensive Geriatric Assessment (AC-CGA) Form and User’s Manual Version 9.3**, 9.3 edn. Washington D.C., USA: interRAI; 2017.

5. Salih SA, Paul S, Klein K, Lakhan P, Gray L: **Screening for delirium within the interRAI acute care assessment system**. *J Nutr Health Aging* 2012, **16**(8):695-700.

6. Travers C, Byrne GJ, Pachana NA, Klein K, Gray L: **Validation of the interRAI Cognitive Performance Scale against independent clinical diagnosis and the Mini-Mental State Examination in older hospitalized patients**. *J Nutr Health Aging* 2013, **17**(5):435-439.

7. Wellens NI, Verbeke G, Flamaing J, Moons P, Boonen S, Tournoy J, Milisen K: **Clinical changes in older adults during hospitalization: responsiveness of the interRAI acute care instrument**. *J Am Geriatr Soc* 2013, **61**(5):799-804.
